# Trust me! Parental embodied mentalizing predicts infant cognitive and language development in longitudinal follow-up

**DOI:** 10.3389/fpsyg.2022.867134

**Published:** 2022-08-04

**Authors:** Dana Shai, Adi Laor Black, Rose Spencer, Michelle Sleed, Tessa Baradon, Tobias Nolte, Peter Fonagy

**Affiliations:** ^1^SEED Center, School of Behavioral Sciences, The Academic College of Tel Aviv-Yaffo, Tel Aviv-Yafo, Israel; ^2^CNWL NHS Perinatal Mental Health Services, London, United Kingdom; ^3^Research Department of Clinical, Educational and Health Psychology, University College London, London, United Kingdom; ^4^Anna Freud National Centre for Children and Families, London, United Kingdom; ^5^School of Human and Community Development, University of the Witwatersrand, Johannesburg, Johannesburg, South Africa; ^6^Wellcome Centre for Human Neuroimaging, University College London, London, United Kingdom

**Keywords:** parental mentalizing, parental embodied mentalizing, cognitive development, language development, coding interactive behavior, emotional availability scale

## Abstract

Children’s cognitive and language development is a central aspect of human development and has wide and long-standing impact. The parent-infant relationship is the chief arena for the infant to learn about the world. Studies reveal associations between quality of parental care and children’s cognitive and language development when the former is measured as maternal sensitivity. Nonetheless, the extent to which parental mentalizing – a parent’s understanding of the thoughts, feelings, and attitudes of a child, and presumed to underlie sensitivity – contributes to children’s cognitive development and functioning, has yet to be thoroughly investigated. According to the epistemic trust theory, high mentalizing parents often use ostensive cues, which signal to the infant that they are perceived and treated as unique subjective beings. By doing so, parents foster epistemic trust in their infants, allowing the infant to use the parents a reliable source of knowledge to learn from. Until recently, parental mentalizing has been limited to verbal approaches and measurement. This is a substantial limitation of the construct as we know that understanding of intentionality is both non-verbal and verbal. In this investigation we employed both verbal and non-verbal, body-based, approaches to parental mentalizing, to examine whether parental mentalizing in a clinical sample predicts children’s cognitive and language development 12 months later. Findings from a longitudinal intervention study of 39 mothers and their infants revealed that parental embodied mentalizing in infancy significantly predicted language development 12 months later and marginally predicted child cognitive development. Importantly, PEM explained unique variance in the child’s cognitive and linguistic capacities over and above maternal emotional availability, child interactive behavior, parental reflective functioning, depression, ethnicity, education, marital status, and number of other children. The theoretical, empirical, and clinical implications of these findings are discussed.

## Introduction

Cognitive and language development are two of the most important achievements made during early childhood – each with long-standing impact on all domains of life. Early cognitive development is associated with the blossoming of memory and establishment of social skills, logical reasoning, planning, and problem solving (Revenson and Singer, 1978; Flavell et al., 2002; Burger, 2010), is predictive of subsequent scholastic achievement (Angrist and Evans, 1998). Language is one of the most important capacities humans acquire which serves cognitive, interpersonal, and social functioning (Madigan et al., 2019), while shaping the child’s thinking and behavior (Xu, 2002), and contributes to communication skills to academic success and lifelong achievement (Stevenson and Newman, 1986; McCardle et al., 2001; Storch and Whitehurst, 2002; Craig et al., 2003; O’Neill et al., 2004; National Institute of Child Health and Human Development [NICHD] Early Child Care Research Network, 2005; Magnuson and Duncan, 2006). As children acquire language, they also become masters at using language to express and serve their needs, which in turn helps them to adjust to and to achieve more in the inherently challenging world (Hoff, 2013).

A central premise in developmental psychology is that the child’s development cannot be studied independently from the parental care the child receives and the interpersonal context in which the child grows up in Belsky (1981); Bronfenbrenner (1992), Sameroff (2009), and Cicchetti (2016). Of the many aspects of parental care, parental sensitivity has been investigated extensively in terms of its impact on child development. Sensitivity has originally been defined as the accurate, prompt, and contingent didactic and affective responses to children’s signals (Ainsworth et al., 1974). According to attachment theory, maternal sensitivity is the foundation upon which the infant’s social, emotional, cognitive, and symbolic development evolves (Bowlby, 1969; Ainsworth et al., 1978). It has further been asserted that disruptions to the formation of maternal sensitivity may interfere with the development of cognitive competencies (e.g., Feldman et al., 2004). Studies have demonstrated the importance of the quality of parental care in early cognitive and language development, showing that more sensitive and responsive parenting predicted infants’ faster rates of both cognitive and language (Hart and Risley, 1995; Brody and Flor, 1997; Tamis-LeMonda et al., 2001; NICHD Early Child Care Research Network, 2003; Raviv et al., 2004; Bradley and Corwyn, 2008; Landry et al., 2008; Newman et al., 2016; Taumoepeau, 2016; Wade et al., 2018). Given the impact of maternal sensitivity on child development, there is value in identifying its antecedents. Low SES, poor health, and lack of family support have been shown to be linked to comprised sensitivity (Cogill et al., 1986; Barker et al., 2013; Agnafors et al., 2019). Also, mental health problems and adversity might undermine sensitive parenting because when negative emotion and maternal difficulties are activated, parents appraise parenting events differently (Dix, 1991). When stress levels are high and the perception of parental abilities are low, parents may be unable to activate the child-oriented emotions that motivate effective caregiving (Booth et al., 2018). Studies with clinical sample also suggest that maternal psychological unavailability and low sensitivity, frequently observed in mothers with mental health difficulties, may have a substantial, deleterious impact on the course of typical developmental attainments (Cicchetti et al., 2000). Noteworthy is that this body of work is modest in comparison to the abundance of work showing how parental care is linked with the child’s emotional and social functioning. Moreover, infants’ cognitive and/or language development has seldomly been examined within the context of maternal sensitivity in a comprehensive, longitudinal format (Feldman et al., 2004; Kertz et al., 2008).

Upon further investigation of the construct of parental sensitivity, there has been increasingly more conceptual and empirical support indicating that the “active ingredient” of maternal sensitivity is parental mentalizing (Meins et al., 2001; Koren-Karie et al., 2002; Kelly et al., 2005; Laranjo et al., 2008). Parental mentalizing refers to a parent’s understanding of the thoughts, feelings, and attitudes of a child (Fonagy et al., 2002; Slade, 2002), and treating the child as an intentional agent (Fonagy et al., 2002). It is believed that sensitive parents use mentalizing to understand their children and to engage with them in line with the child’s needs, wishes, feelings, and thoughts (Krink et al., 2018). Indeed, parental mentalizing has been shown to play an important role in children’s socio-emotional development, including attachment security and social cognition, often also when controlling for maternal sensitivity (Fonagy et al., 1991; George and Solomon, 1999; Meins et al., 2001, 2002; Koren-Karie et al., 2002; Slade et al., 2005; Sethna et al., 2012; Shai and Belsky, 2017; Shai and Meins, 2018; Gagné et al., 2021).

Parental mentalizing appears to be compromised by parents’ mental health problems, especially when coupled with high levels of social adversity (Bateman and Fonagy, 2001). These clinical populations will inevitably exhibit some difficulties with mentalizing because the combination of parental mental health problems and socioeconomic disadvantage can have deleterious effects on parent child relationships and child development (Fonagy et al., 2016). Indeed, mothers with mood and anxiety disorders often had difficulty responding appropriately to infant cues, while exhibiting both under responsiveness and over-responsiveness interactive behaviors (Diego et al., 2006; de Camps Meschino et al., 2016). Others have found that maternal depression symptoms, such as low mood, negative thoughts, and attributions regarding her infant, may reduce a mother’s ability to mentalizing, and thereby interfering with her ability to be adequately responsive to her infant’s cues (Cicchetti and Toth, 1998; Reck et al., 2004).

In contrast to the body of work examining links between parental mentalizing and children’s socio-emotional development, the extent to which parental mentalizing contributes to children’s cognitive or language development and functioning has yet to be investigated. What might be the mechanism linking parental care and cognitive development? One explanation for this established link is that parental responsive and sensitive caregiving provides the infant with a sense of safety and reassurance to explore and investigate the world, thereby promoting the cognitive and language capacities (Madigan et al., 2019). Moreover, it is believed that a sensitive and responsive parent is more likely to operate within the child’s zone of proximal development (Vygotsky, 1967), thereby strengthening and developing the foundations cognitive and language functioning (Meins, 1997). In fact, it has been argued that the combination of parental care that is characterized with contingent and sensitive responsiveness, together with cognitive responsiveness to the child’s needs, including the provision of rich verbal input and maintaining and expanding on the child’s interests, provide a solid supportive foundation necessary for various aspects of a child’s learning (Smith et al., 2006; Landry et al., 2008).

Nonetheless, there has been no specific or detailed model to suggest why and how the quality of parental care and cognitive/language development may be linked. The recently conceived model of epistemic trust may be useful in this context, as it offers an integrated framework linking parental mentalizing, epistemic trust, social learning, and ultimately cognitive development. Essentially, the theory of epistemic trust creates a conceptual link between the child’s emotional relationship with an adult and their openness to information conveyed to them by that person, including their capacity to assimilate the information communicated (Fonagy and Allison, 2014; Fonagy et al., 2015, 2019). According to this framework, social learning is a function of the epistemic trust the “learner” (child) has for the “instructor” (parent).

The theory of natural pedagogy holds that cues of ostension open a channel protected by evolution for the rapid and efficient transfer of culturally relevant information (Gergely and Csibra, 2005; Csibra and Gergely, 2009, 2011; Kiraly et al., 2013). Ostensive cues refer to certain signals – verbal and non-verbal – that are employed by an agent to prepare the addressee for the agent’s intent to communicate and that the receiver is the addressee of a communication. Examples of ostensive cuing include making eye contact, accurate turn-taking, appropriate and contingent (in time, tone, and content) reactivity, and frequent use of a special communicational tone that addresses the child’s experiential world. All such cues appear to trigger a special mode of learning in the child (Gergely and Király, 2004; Csibra and Gergely, 2011), and create a foundation to acquire further knowledge from that caregiver (Egyed et al., 2013). Indeed, and based on the theory of natural pedagogy, Fonagy and colleagues (Fonagy and Allison, 2014; Fonagy et al., 2015, 2019) argued that any communication marked by the recognition of the listener as intentional agent – via the use of ostensive cues – will reduce epistemic vigilance and increase epistemic trust and the likelihood of communication being coded as relevant, generalizable, and to be retained in memory as important in other contexts.

The importance of caregivers’ mentalizing is apparent in the significant *emotional role* ostensive cues play during the learning interaction. They convey to the communication recipient that he or she is recognized as a subjective, agentive self, “visible” entity, which has a distinct world from their surrounding (Tomasello, 1995; Mayes et al., 2007; Gross, 2010; Csibra and Gergely, 2011). It could be argued that ostensive cues are a behavioral manifestation of parents’ mentalizing capacities (Fonagy and Allison, 2014). Thus, parental mentalizing, in which the parent recognizes and appreciates the child’s uniquely subjective world and internal states, is likely to lead the parent to use ostensive cues more often and more accurately when communicating and teaching the child about the world, people, feelings, and his or herself. On the child’s end, in turn, feeling contingently (sensitively) responded to, seen, and understood – emerging from the adult’s mentalizing stance – imbues in the child a sense that this person can be trusted. After all, if this adult knows me, understands me, and sees me, maybe they also hold important and accurate knowledge about the world, knowledge I now want to learn from them. Under such circumstances, the adult’s mentalizing stance may become a possibly general ostensive cue signaling to the child that epistemic vigilance can be relaxed, and therefore a primary biological signal that it is safe to learn.

We might expect then that those infants cared for by parents with higher mentalizing would show more rapid or advanced language and cognitive development. Indeed, recent research supports this speculation and shows that ostensive cues are involved in meaningful general social interaction and play a key role in supporting learning of language (Axelsson et al., 2012), cognition (Frith, 2008), as well as socio-emotional development (Gergely, 2007; Senju and Csibra, 2008). Nonetheless, there is yet little research linking ostensive cuing, parental mentalizing, and child learning capacities.

Reexamination of the literature linking parental mentalizing and sensitivity to child cognitive and language development reveals the limitations of these studies since examinations of the effect of verbal sensitivity or parental mentalizing on verbal development is confounded by the overlap of modality. In other words, being treated sensitively or being mentalized by parents may advance infant development just because of the language advantage it brings, and thus manifests the advantage of a richer language environment and may not be related to mentalizing opening the child’s mind to social learning. Moreover, reliance on verbal and linguistic exchanges between parents and infants posits a substantial limitation of the constructs, as we know that understanding of intentionality in human communication is, at least initially non-verbal, and continues to be both verbal and non-verbal as human interactions and mentalizing increases in complexity (Malle, 2021).

The current study is a part of a program of work which intends to extend mentalizing beyond the uniquely verbal modality to also include the non-verbal bodily interactions of parents and infants (Shai and Belsky, 2011a,Shai and Fonagy, 2014; Shai and Belsky, 2017; Garset-Zamani et al., 2020; Væver et al., 2020; Gagné et al., 2021; Afek et al., 2022). This is reflected in the more recent operationalization to parental mentalizing, namely, Parental Embodied Mentalizing (PEM; Shai and Belsky, 2011a,Shai and Belsky, 2017), which is a *non-verbal*, bodily-based, measure of the parent’s capacity to envision the child and respond to him or her as a psychological and subjective agent. The construct and measure of PEM is based on the premise that mental states are expressed also through the quality of body movements, and PEM attempts at capturing the caregiver’s ability to make sense of the baby’s body-based signals and quality of movement accompanying them in terms of their underlying mental states. This approach focusses solely on the non-verbal exchanges taking place between the adult and the infant, while viewing and coding pre-recorded video of parent-infant interactions with the sound turned off.

The parent’s embodied appreciation and implicit representation of, and adaption to the infant’s mind is assessed by parameters such as the velocity, shape, distance, or direction of movement of both the parent and the infant. The conceptual and operational use of PEM and its movement-based “language” affords assessing the degree to which the caregiver responds appropriately to the infant’s mental states through the movement-based interaction between them (Væver et al., 2020). Parents with high PEM capacities prove capable of modifying faster and more accurately their own movement patterns in response to their interactive failures and the infant’s signaling, so that they respond more accurately to the infant’s non-verbally manifested mental state. In contrast, parents with poor PEM capabilities are less likely to modify flexibly their quality of the movements, and tend to fail to detect, or misinterpret, the infant’s embodiedly manifested mental states, resulting in responses that contradict the infant’s mental state (Shai and Belsky, 2017).

## The current study

Appreciating the importance of cognitive and language development in infancy for functioning in all life domains, this work aims at further illuminate what factors may be involved in individual differences in infants’ cognitive and language development. To illuminate this process, the theoretical framework of epistemic trust is useful, as it argues that when the infant perceives the caregiver to be trustworthy, he or she is much more likely to learn from the caregiver. This trust is achieved by the caregiver signaling to the infant that he or she is seen and appreciated as intentional being via the use of ostensive cuing. In this sense, parental embodied mentalizing may be a robust way for the parent to communicate to the infant ostensive cues, by indicating to the infant’ on the bodily level – that is so accessible and prominent for him or her – that he or she is responded to their internal worlds based on what they are communicating to the parent through their bodily movements and the quality of their movements. It is possible that this powerful ostensive cueing, in turn, will reduce ostensive vigilance and open the highway to learning. Therefore, we contend that the understanding through body-based, verbal-free, interaction is important in establishing epistemic trust as it represents to the infant powerful ostension, making them more open to social learning. Hence, this study aims at testing the possible contribution of parental mentalizing as means to foster epistemic trust in the infant, evident in higher cognitive or language development.

As mentioned, the relationship between parental sensitivity, mentalizing, ostensive cues, and infant cognitive and language development is important and meaningful in all populations, and holds particular interest and importance in clinical populations (Fonagy et al., 2016). Therefore, the current work focuses on parent-infant dyads at risk. Importantly, previous work on this sample did not reveal group differences in the child’s cognitive or language development over time (Fonagy et al., 2016); therefore, the aim of this work was to put to test the hypothesis that parental mentalizing – verbal or embodied – can, when controlling for group and parental sensitivity, predict children’s cognitive and language functioning over a 12-month period.

## Measures and methods

### Participants

The current study draws on a randomized controlled trial including families experiencing high levels of social adversity and maternal mental health problems and investigated the effect of parent-infant psychotherapy relative to treatment as usual (such as health visiting, general practitioner, and access to psychology therapies). The families included in the study experienced socioeconomic disadvantages and living inner city demographically diverse areas. A family was referred to the study if an independent professional identified the mother to suffer with mental health illness and was the caregiver of their baby under 1-year old. Maternal Social Exclusion Criteria were assessed on the following: low-Income household, long-term unemployed temporary/crowded, accommodation, single-parent household, chronic illness or physical disability, childhood foster/institutional, care social isolation (recent relocation), <20 years of age, previous diagnosis of psychiatric illness and number of social exclusion criteria met (Fonagy et al., 2016).

Of the 76 participants who consented to take part in the study, 39 participated in the current investigation. Attrition was due to missing video recorded parent-infant interactions (refusal to consent to be video recorded, or technical limitations), or missing data in the 12-months follow-up due to difficulty engaging the family.

### Recruitment

The study took place at four sites in England; all identified as serving demographically diverse, urban populations with areas of high levels of socioeconomic deprivation. The sites were three hospital-based perinatal psychiatry units and one community children’s center. Referrals to the study were made by professionals working in health and social care (e.g., health visitors, psychiatrists, and children’s center workers). Reasons for referral were maternal mental health difficulties (100%), mother and infant attachment bonding difficulties (27.7%), domestic abuse of marital problems (12.3%), maternal physical problems including (4.6%), social problems including loneliness, isolation, lack of family support and financial and social problems (18.5%), bereavement of trauma (6.2%). Please see Table 1 for further information about the sample.

**TABLE 1:** Demographic variables for the intervention and control groups.

	Control group (*n* = 15)			Intervention group (*n* = 24)			Intervention vs. control	
	*Range*	*M*	*SD*	*Range*	*M*	*SD*	*t (df)*	*P*
Infant age at T1 (months)	0.6–10.6	3.55	3.11	0.50–9.4	3.63	2.81	−0.08 (37)	0.94
Infant age at T2 (months)	12.9–27–5	17.23	4.23	12.9–22.4	16.13	2.8	0.97 (36)	0.34
	n	%		n	%		χ*2 (df)*	*P*
Infant sex: Male	12	80		17	71		0.41 (1)	0.52
Maternal ethnicity: White	8	53		17	71		1.23 (1)	0.27
Maternal marital status: Married/cohabiting	8	53		15	62		0.32 (1)	0.57
Maternal education: Higher education	11	73		9	38		4.7 (1)	0.03
Maternal social exclusion criteria								
Low-income household	4	27		13	54		2.84 (1)	0.09
Long-term unemployed	3	20		5	21		0.00 (1)	0.95
Single-parent household	7	47		8	33		0.69 (1)	0.41
Chronic illness or physical disability	15	100		2	8		1.32 (1)	0.25
Childhood foster/institutional care	15	100		1	4		0.67 (1)	0.41
Social isolation (recent relocation)	3	20		10	42		1.95 (1)	0.16
<20 years of age	15	100		0	0		0	0
Previous diagnosis of psychiatric illness	10	67		16	67		0.00 (1)	1.00

Maternal ethnicity: Control group – White 53%, Black 26%, Asian 7%, Mix race 7%, Arabic 7%. Intervention group – White 71%, Black 8%, Asian 13%, Mix race 4%, Arabic 4%.

### Ethical approval

The study received ethical approval from the National Health Service REC (Reference: 05/Q0511/47) and registered on the International Standard Randomized Controlled Trial Number Register (ISRCTN38741417).

### Treatment and control groups

Parent-infant dyads were randomly allocated to control (*n* = 15) or intervention (*n* = 24). A constructive treatment strategy was adopted in the selection of the comparison condition (Kazdin, 2002): All participants received standard treatment (Fonagy et al., 2016). There were no significant differences between the two groups in the number of contacts with health, social care, and mental health services that families used during the study period, apart from a slightly higher number of general practitioner contacts for mothers in the PIP group at the 6-month follow-up relative to controls (Fonagy et al., 2016). The control group mothers received routine care, including the standard health and social care services available to them, such as health visitors, GPs, psychiatrists, counselors, psychologists, family support workers, community mental health team, child psychologists, and psychotherapists. Significant input from secondary or specialist primary care. Dyads in the Parent-Infant Psychotherapy treatment group were invited to attend appointments with a parent–infant psychotherapist. The model of intervention was manualized (Baradon et al., 2005) and provided by six experienced parent–infant psychotherapists. The clinicians implementing the intervention were among those who developed the model and its manualization, and thus were familiar with the nuances of implementation. The team had 2-weeks’ group supervision so that clinical practice was discussed in depth and shared among the clinicians to ensure model adherence. It is not a model that follows prescribed sessional topics or patterns, so adherence could not be measured explicitly (Fonagy et al., 2016). In the sessions, parents raise any matter on their mind concerning their own mental/feeling state, factors that are affecting it, their relationship with their infant, and issues about the infant. The therapist will focus on observing interactions in the room and trying to understand and make meaning of them. The therapist attention is given to non-verbal communications and communication errors which are associated with disorganized attachments. The baby is in the center of the intervention, with the aim of addressing precocious defensive behaviors (e.g., avoidance, inhibition, and dissociation) (Fonagy et al., 2016).

### Measures and procedure

Following referral to the study, potential participants were screened for inclusion and exclusion criteria. Informed consent was sought from all eligible participants and baseline assessments were carried out (prior to randomization, T1). Participants were randomly allocated to one of the two groups (treatment as usual or treatment group), and follow-up assessments were carried out 12 months post-randomization (T2). Assessments took place in the families’ homes and in the local clinics from which they were referred. During each assessment, semi-structured interviews were conducted with the mothers, to later be coded for parental mentalizing, mothers were asked to complete several questionnaires, a developmental assessment was carried out with the babies, and 10-min video recordings were made of parent–infant free-play interactions, later coded used of three different coding systems – CIB, PEM, and EAS. For the video-recorded interactions, mothers were asked to “spend time with your baby as you usually would.”

### Maternal psychopathology

#### Maternal depression

The Center for Epidemiological Studies Depression Scale (CES-D; Radloff, 1977) was used to assess maternal depression. The CES–D consists of 20 items in a self-report format measuring depressive symptoms experienced in the past week on a four-point Likert scale ranging from 0 (‘rarely or none of the time’) to 3 (‘most or all the time’). Example items include “during the past week I was bother by things that don’t bother me” and “I thought my life had been a failure” (Radloff, 1977). Scores range between 0 and 60 with higher scores indicating greater depressive symptoms.

### Maternal sensitivity measures

#### Emotional availability scale

Biringen et al. (1993) the EAS was used to assess the video-recorded dyadic interaction for the emotional availability of the parent to child and child to the parent. Emotional availability refers to a person’s ability to express their emotions and to perceive and respond to the emotional needs and goals of another (Emde, 1980). The EAS consist of four parental scales: sensitivity, structuring, non-intrusiveness and non-hostility, well as two child scales: Child Responsiveness and Child Involvement. In the current study, we used the Emotional Availability Sensitivity scale.

#### Coding interactive behavior

Feldman (1998) the CIB rating scales were used to code the video-recorded parent-infant interactions, and in the current study, the *dyadic attunement* scale was used as an index of sensitivity. This scale reflects an overall mutuality between parent and infant. The factor relates to an interaction where the parent is sensitive, non-intrusive, consistent, and supportive. There is no tension or constriction, and the interaction is reciprocal and well adapted to the affective state of each partner. It is the sum of 11 items, with a potential range of 11–55.

### Parental mentalizing measures

#### Parental reflective functioning

Slade et al. (2004) was measured using the Addendum to the Reflective Functioning Scoring Manual (Slade et al., 2004) developed for specific use with the Parent Development Interview-Revised-Short Form (PDI-R2-S; Slade et al., 2004). The PDI is a 35-item semi-structured interview that taps into parental representations of themselves as parents, their child, and the relationship. Questions are designed to capture parents’ understanding of their own and their child’s thoughts, feelings, and behaviors. The PDI transcripts were coded for parental reflective functioning using the parental RF scale, which ranges on a 11-point scale ranging from -1 (negative RF) to 9 (full or exceptional RF), where scores below five represent negative, absent, or low RF, and scores of five and above represent moderate to high levels of RF. Interviews were transcribed verbatim and coded by trained raters on the PRF coding system.

#### Parental embodied mentalizing

Shai and Belsky (2017) was used to assess parental embodied mentalizing. PEM measures (a) the parent’s capacity to implicitly conceive, comprehend, and extrapolate the infant’s mental state from the whole-body kinesthetic expression of the baby; and (b) the parent’s ability to adjust their own kinesthetic patterns accordingly (Shai and Belsky, 2011a,Shai and Belsky, 2017). Specifically, PEM assesses the degree to which the parent can appreciate the infant has mental states that motivate his or her behavior, and that these mental states are expressed embodiedly. This parental appreciation is assessed via his or her bodily adjustment and attunement to the infant’s movement. The appropriateness and accuracy of the parental attunement is determined through the infant’s response to the parental attempt at responding to the infant. In other words, the infant is the one who informs the coder to determine the extent to which the parent’s embodied response suited the infant’ mental state. To that end, coding PEM involves exclusive focus on dyadic bodily-based movements. Video-recorded interactions are rated on mute-mode, with the goal of avoiding potential influences that verbal input may have in evaluating the kinesthetic (bodily) interaction. The video is run on normal speed, but frequently paused and played back to view the interaction frame-by-frame. PEM is coded in four stages and rated on a nine-point scale. Very low PEM scores indicate that the parent demonstrates severe difficulty in acknowledging the infant as a mental entity. Very high PEM scores indicate the parent can detect even subtle mental states and repair interactive ruptures very quickly (Shai and Belsky, 2017).

Cognitive and language development were assessed using the Bayley Scales of Infant Development, Third Edition (BSID-III; Bayley, 2006). The Bayley-III assessment reflects current federal, state, and professional standards for early childhood assessment, and is administered by a trained and experienced evaluator. The Bayley-III *Cognitive Scale* includes items that assess sensorimotor development, exploration and manipulation, object relatedness, concept formation, memory, and additional aspects of cognitive processing. The Bayley-III *Language Scale* provided a focus on prelinguistic behaviors, reasons for communicating, and social routines, as well as both expressive and receptive language skill. Bayley (2006) reports excellent split-half reliability (between 0.71 and 0.97) and internal consistency (between 0.74 and 0.99) and adequate test-retest reliability (between 0.69 and 0.86). Studies have shown good concurrent validity with other measures of physical and cognitive functioning (Provost et al., 2000; Voigt et al., 2003).

## Results

### Data analysis plan

To test the hypotheses of the current study, we first examined whether the two groups differed from one another in the background and/or parental care variables, namely, emotional availability sensitivity, CIB attunement, parental verbal mentalizing, and parental embodied mentalizing. Next, we ran zero-order correlations to examine possible associations between background and study variables. Finally, we tested the study’s central hypotheses that parental care constructs would predict cognitive and language development using two stepwise linear regressions in SPSS 27 (IBM SPSS Statistics for Macintosh, Version 27.0).

A shown in Table 1, results revealed no group differences in background variables between the intervention and the control group, with one exception of the treatment group being significantly more educated than the control group. Therefore, we included this variable in all our further analyses. In terms of parental care variables, no difference was revealed between the intervention and the control group in terms of parental sensitivity or mentalizing measures at T1. Specifically, there were no differences between the intervention and control groups in Emotional Availability (EAS) Maternal sensitivity scale [*t*(58) = 0.51, *p* = 0.61], CIB Dyadic attunement [*t*(58) = 0.22, *p* = 0.83], Parental Reflective Functioning (RF) [*t*(73) = −1.16, *p* = 0.25] or Parental Embodied Metalizing (PEM) [*t*(57) = −0.38, *p* = 0.70] between those allocated to the control or the intervention groups.

Since no group differences were detected in baseline levels of the parental care variables, the next step involved examining associations between background and parental care variables. As shown in Table 2, the background variables of child sex, marital status, and postnatal depression (CES-D) did not correlate significantly with any of the parental care variables. Maternal Ethnicity was positivity correlated with PRF and PEM, such that white mothers had higher PRF and PEM scores. Number of other children was negatively correlated with PRF, such that the more children’s mothers had, and the younger the child was in the family order, the lower mothers’ RF was. Maternal education was significantly related to CIB, EAS, and PEM, such that the more education mothers had, the higher they were rated on CIB Attunement, EAS sensitivity, and PEM. With respect to associations between the parental care variables, results revealed that PEM correlated positively with both CIB and EAS sensitivity, such that mothers with higher PEM were also rated as more sensitive both on the CIB and the EAS scales.

**TABLE 2:** Means, standard deviations, and zero-order correlations between background variables and research variables.

	1	2	3	4	5	6	7	8	9	10
1. Maternal ethnicity	–									
2. Child sex	−0.01	–								
3. Marital status	−0.15	0.20	–							
4. Number of other children	−0.27*	0.15	0.11	–						
5. Maternal education	0.16	−0.20	−0.15	−0.11	–					
6. CES-D	−0.11	−0.01	−0.13	0.18	−0.43**	–				
7. CIB attunement	0.20	0.05	−0.03	0.12	0.36**	−0.02	–			
8. EAS sensitivity	0.08	−0.06	0.02	0.02	0.28*	−0.18	0.59**	–		
9. PRF	0.25*	0.03	0.03	−0.42**	0.17	−0.14	0.12	0.11	–	
10. PEM	0.41**	−0.18	0.09	−0.11	0.35**	−0.21	0.37**	0.27*	0.11	–

*p < 0.05, **p < 0.01. CIB Attunement, coding interactive behavior; EAS, emotional availability, maternal sensitivity scale; PRF, parental reflective functioning; PEM, parental embodied mentalizing.

Applying a stepwise linear regression analysis, and as shown in Table 3, results revealed that PEM at T1 marginally predicted child cognitive development 12 months later (T2) over and above maternal EAS, CIB attunement, PRF, maternal ethnicity, education, number of children and maternal depression [*F*(9,30) = 1.88, *p* = 0.09]. Moreover, applying a second stepwise linear regression analysis, and as shown in Table 4, revealed that PEM at T1 predicted child language development 12 months later (T2) over and above maternal EAS, CIB attunement, PRF, maternal depression, ethnicity, education, and number of children [*F*(9,28) = 2.3, *p* < 0.005].

**TABLE 3:** Standardized regression coefficients for infants’ cognitive development scores regressed on background variables.

	Step 1					Step 2				
	β	*SE*	*p*	95_*Lo*_	95_*Hi*_	β	*SE*	*p*	95_*Lo*_	95_*Hi*_
Maternal ethnicity	0.05	4.26	0.78	−7.47	9.93	−0.02	4.36	0.89	−9.50	8.33
Maternal education	0.11	1.46	0.53	−2.05	3.91	0.06	1.46	0.73	−2.47	3.50
Number of other children	−0.06	2.88	0.75	−6.81	4.92	−0.07	2.82	0.72	−6.78	4.76
CES-D	0.14	0.18	0.41	−0.22	0.52	0.17	0.18	0.30	−0.18	0.56
Group	−0.34	3.99	0.04	−16.60	−0.31	−0.38	3.96	0.03	−17.34	−1.18
PRF	0.26	1.68	0.13	−0.80	6.04	0.25	1.65	0.13	25.42	5.94
CIB Attunement	0.30	0.40	0.11	−0.17	1.48	0.27	0.40	0.15	−0.22	1.41
EAS maternal Sensitivity	−0.04	1.57	0.84	−3.52	2.89	−0.06	1.55	0.73	−3.70	2.63
PEM						0.25	2.31	0.15	−1.31	8.13
*R* ^2^	0.31					0.36				
Adjusted *R*^2^	0.14					0.17				

Group, control or intervention; PRF, parental reflective functioning; CIB Attunement, coding interactive behavior; EAS Maternal Sensitivity, emotional availability; PEM, parental embodied mentalizing.

**TABLE 4:** Standardized regression coefficients for infants’ language development scores regressed on background variables.

	Step 1					Step 2				
	β	*SE*	*p*	95_*Lo*_	95_*Hi*_	β	*SE*	*p*	95_*Lo*_	95_*Hi*_
Maternal ethnicity	−0.13	4.88	0.51	−13.27	6.70	−0.26	4.36	0.14	−15.60	2.27
Maternal education	−0.24	1.63	0.23	−5.32	1.33	−0.35	1.44	0.05	−5.94	−0.03
Number of other children	0.16	3.19	0.44	−4.01	9.02	0.16	2.77	0.39	−3.25	8.07
CES-D	−0.21	0.20	0.26	−0.63	0.18	−0.14	0.17	0.39	−0.50	0.20
Group	0.14	4.42	0.45	−5.65	12.42	0.08	3.86	0.63	−6.03	9.79
PRF	0.20	1.88	0.28	−1.79	5.88	0.18	1.63	0.27	−1.52	5.16
CIB attunement	0.41	0.46	0.07	−0.06	1.83	0.33	0.41	0.09	−0.11	1.55
EAS maternal sensitivity	−0.05	1.83	0.80	−4.21	3.29	−0.08	1.59	0.65	−3.99	2.54
PEM						0.53	2.23	0.00	2.66	11.79
*R* ^2^	0.21					0.43				
Adjusted *R*^2^	−0.01					0.24				

Group, control or intervention; PRF, parental reflective functioning; CIB Attunement, coding interactive behavior; EAS Maternal Sensitivity, emotional availability; PEM, parental embodied mentalizing.

## Discussion

This longitudinal study aimed to expand existing knowledge on the importance of parental care on child development and to test the hypothesis that parental care, namely, two modes of parental mentalizing in a clinical sample can predict infant cognitive and language development 12 months later when controlling for parental sensitivity measures, maternal depression, background variables, and assignment to clinical treatment or control group.

First, we examined whether mothers in the treatment group differed from mothers in the control group in terms of their parental care at the beginning of the study. We detected no group differences in parental care variables: sensitivity measures (emotional availability and CIB attunement), or parental mentalizing measures (PEM and parental RF). Additionally, as mentioned earlier, Fonagy et al. (2016), found no differences in cognitive or language abilities between those infants who participated in parent-infant psychotherapy and those who were in the control group. These null findings could be attributed to the variance of the sample, including relatively high attrition rates (18 and 32% in the PIP and control groups, respectively), as well as variability in number of sessions offered, taken, and missed. A considerable proportion of the participants randomized to the treatment group attended only one or no sessions (*n* = 7, 18%), and more than one third of the dyads assigned to the treatment group attended fewer than five sessions (*n* = 14, 37%). Fonagy et al. (2016) reported that when the dyads who did not attend any sessions were excluded from their analysis, the results did not differ from those reported when following the intent- to-treat approach to the analysis. Nonetheless, they assert, the fact that many dyads in the treatment group received very little therapeutic input may dilute the treatment effects for those mothers and babies who engaged successfully in the treatment. Clearly, it is also possible that because of the small sample size (*n* = 76), the study lacked power to detect treatment effects.

Considering these results revealing no group differences in the outcomes, the central question in the current study was the extent to which parental verbal or embodied mentalizing can predict child cognitive and language development 12 months later when controlling for gold standard parental sensitivity measures, namely emotional availability sensitivity and CIB attunement. Results showed that of all the examined parental care variables, only parental embodied mentalizing (PEM) at T1 marginally predicted child cognitive functioning 12 months later, and significantly predicted child language development after 12 months (T2). In both these models, PEM predicted the child’s outcomes over and above maternal EA sensitivity, CIB attunement, parental reflective functioning, maternal ethnicity, education, group, number of children and maternal depression. In other words, PEM explained unique variance in individual differences detected over time in children’s language and indicated the same to apply at trend level for cognitive development when considering traditional and well-established measures of parental care, parental depression, and a variety of parental background variables that theoretically could be associated with parental mentalizing capacities.

In terms of the theory of epistemic trust (Fonagy and Allison, 2014; Fonagy et al., 2015, 2019), these findings support the assertion that parental mentalizing plays a role in the children’s cognitive development and growth. It is conceivable that repeated experiences with a high PEM parent fosters in the baby a sense of being heard, seen, understood, and appreciated for whomever he or she is–being considered as an intentional agent. Although our design did not allow for testing this as a mechanism, PEM- and thereby body- or kinesthetically conveyed mentalizing, may create interpersonal modes of shared attention and experience that are developmentally appropriate, and which are linked with better infant learning. In other words, a parent with high PEM may very well be making frequent and appropriate use of ostensive cues to the infant.

The question remains as to why only PEM, an embodied approach to parental mentalizing, and not verbal parental mentalizing, predicted children’s cognitive and language development in this study and in this population? PEM may be capturing the parent’s optimal way to communicate to the non-verbal infant that they are treated as psychological agents, using the “interpersonal language” infants master and are familiar with, a language that makes most sense to them. In this way, it is tenable that a parent with high PEM communicates with the infant using abundance of ostensive cues that are accessible and meaningful to the infant, thereby communicating effectively to the infant that epistemic vigilance can be calmed and invite to establish epistemic trust.

The results of the current investigation are also suggestive of the mechanism through which parental embodied mentalizing scaffolds learning in general, and particularly language acquisition. Both language and mentalizing assume a representational capacity, where one thing is treated as something else (Fonagy and Target, 2006; Halfon and Bulut, 2019). High PEM capacity is reflective of the parent’s appreciation that the infant’s physical actions and their quality express the infant’s subjective experience and internal world. Thus, the parent may be constantly engaged in a symbolic act, inferring from action and movement what the infant is feeling, experiencing, and communicating. Over repeated such interpersonal experiences, where the infant experiences that his or her mental states and subjective experience are symbolized to him or her, the infant can gradually internalize this embodied act of symbolization, which lays the foundations for the symbolic action inherent in language.

When, in contrast, the infant’s physical states are not treated as expressions of his or her internal world, the infant may experience a disconnect between body and mind and fail to learn about the meaning or the realness of his or her mental states. In this way, the concept of symbolization is less embedded in the infant’s experience, which in turn can lead to slower rates of language development.

The possible usefulness of PEM exhibited in the current investigation could further be illuminated by distinguishing between two general modes of processing, and particularly mentalizing. Most of our daily interactions – especially in the domain of parent-infant interactions – involve implicit and pre-reflective rather than explicit and reflective mentalizing (Allen et al., 2008; Shai and Belsky, 2011b). Explicit mentalizing comprises a relatively slow process, which is typically verbal and requires reflection, awareness, and (Satpute and Lieberman, 2006; Allen et al., 2008). As such, it is likely to be captured by the measurement of parental reflective functioning. In contrast, implicit mentalizing involves faster processing and it is typically reflexive and requires little or no awareness or effort (Satpute and Lieberman, 2006; Allen et al., 2008), as captured by the PEM coding system.

These two types of mentalizing – implicit and explicit – are specific cases of types of knowledge and representation. The former is underpinned by semantic knowledge, which relies on symbolic representation in language, while the latter is based upon procedural representation (Kihlstrom and Cantor, 1984). Based on developmental research (e.g., Ainsworth et al., 1978; Tronick, 1989; Beebe and Lachmann, 1994), the Process of Change Study Group (Lyons-Ruth et al., 1998), content that one important form of procedural knowledge pertains to how to do things and be with others. The group has termed this as “implicit relational knowing,” emphasizing that such “knowings” are as much affective and interactive as they are cognitive. According to the researchers, this implicit relational knowing begins to be represented in some yet to be known before language is available and continues to operate implicitly across the life span. Moreover, implicit relational knowing typically operates outside focal attention and conscious experience, without benefit of translation into language. Specifically, language can be used in the service of this knowing but the implicit knowings governing intimate interactions are not language-based and are not usually translated into semantic form (see also Shai and Belsky, 2011b).

Hence, a parent with high PEM may be providing the infant with developmentally appropriate suitable and long-lasting interpersonal communicative building blocks, the principles, or the infrastructure of communication skills, long before the baby expresses explicit verbal language (Wade et al., 2018). More specifically, infants and parents successively and jointly bridge the non-verbal gap and create a shared affective and phonological “alphabet” and common ground in relation to a growing intersubjective understanding of affects and intentions, needs and motives, joint attention, and shared experience with the environment (Papoušek, 2007; Adamson, 2018). In this way, a parent with high PEM can establish a meaningful relationship between the parent and the baby that becomes the context for acquiring and elaborating all subsequent communication skills (Quevedo et al., 2012).

What is the experience of an infant with a parent with high PEM? The everyday and repeated interactions with such a parent provide a relational platform in which the infant learns that their behavior – which varies in relation to their behavioral and affective states, interests, and motives – is contingently and differentially answered, mirrored, and understood by a main caregiver (Papoušek, 2007). Through these affectively contingent interactions, infants learn to use their facial or vocal behavior in an instrumental and directional manner, to solicit anticipated contingent answers from their parents (Stern, 1999). This communicative foundation, in turn, is likely to foster a sense of epistemic trust in the parent, allowing the infant to learn from the parent in an effective and rich way (Fonagy and Allison, 2014).

### Study limitations and future research

The findings of this study contribute to existing literature pertaining to the importance of emotional interpersonal processes in infancy to subsequent cognitive and language development, with emphasis on the unique importance of parental embodied mentalizing. Nonetheless, several limitations of the current investigation merit consideration. Firstly, the sample in the current investigation was small (*n* = 39) and included a clinical population of mothers with mental health problems who also were experiencing high levels of social adversity and their young infants. A larger sample size would possibly allow the demonstration of more robust effects, that could also be generalized to a broader population. It is not impossible that in a low-risk sample also verbal measures of parental care would have been detected. Thus, replication studies would benefit from including a larger, low-risk sample, and comparing it to the current findings pertaining to the high-risk sample, thereby providing a possibly more accurate picture of the nature and strength of association among these variables.

Secondly, one of the confounding variables detected in this study was the ethnicity of the mothers, such that Caucasian mothers were rated higher on PEM than mothers who were non-white. Noteworthy is that the PRF and PEM data were analyzed by Caucasian research assistants. Accordingly, it is impossible to outright disregard the possibility that there was a cultural bias in coding parental verbal and embodied mentalizing. Clearly, it is pivotal to conduct replication studies involving coders from various ethnic backgrounds, as well as comparing PEM and PRF across different ethnic groups. Lastly, the present study focused solely on parental care in mothers. In exploring the lines of enquiry for future research discussed above, it is important to also include fathers and examine if they follow the same pattern of findings or do their parental care predict differently the child’s cognitive and language development.

Thirdly, this study’s results support the theory of epistemic trust and are interpreted considering it. We have speculated that parents with high PEM use more frequently and more suitably ostensive cues which in turn foster in the infant epistemic trust and the propensity to use the parent as a trustworthy agent from whom to acquire information and knowledge. Nonetheless, the current investigation did not assess parental use of ostensive cues directly. The field would benefit from this caveat been addressed in future studies, thereby further illuminating the mechanisms through which emotional interpersonal ties impact the human cognition.

### Clinical applications

Given the importance of child language development to subsequent functioning in all domains of life (Madigan et al., 2019), there is a considerable body of clinical work devoted to enhancing and supporting language development. Interventions aimed at reducing language development gaps and promoting language in younger children often focus on encouraging parental talk through parent-child interaction (Canfield et al., 2020), often through encouragement of parental reading aloud and parent–child book sharing (Reese et al., 2010; Aram et al., 2013), thereby enhancing maternal talk to the infant (Canfield et al., 2020). It has been maintained that storytelling is an effective pedagogical instrument in the development of language skills (Cameron, 2001; Isbell et al., 2004), being memorable to learners, helping them learn and retain vocabulary, grammatical structures, and pronunciation (Wajnryb, 2003).

Although increasingly popular, the findings of the current work tentatively suggest that focus on parental book reading and sharing with the infant alone may be insufficient, and that the quality of such interactive exchanges is pivotal. Our preliminary findings indicate that parental embodied mentalizing predicted language development even when accounting for other, gold standard, maternal care constructs, as well as mothers’ ethnicity, number of children, marital status, depression, or education. That is, what might be central to the child’s language development is not the mere existence and practice of story sharing activity with the parent, but the quality of such an activity. In other words, what might be crucial to the success of story-reading parent-infant interactions in terms of language development is that the child’s experiences a parent who is attentive and response to their embodiedly expressed mental states, including following the child’s cues as for his or her focus of interest, attention span, interpretation and playfulness with the story, the images, or the book itself. Moreover, higher PEM ratings indicate higher capacity to repair interactive, dyadic ruptures. It is true that all parents do not always or automatically know what needs or desires their infant is expressing; but those with high PEM capacities prove more capable of and faster in modifying their own kinesthetic patterns in response to their failures so that they respond more accurately to the infant’s non-verbally manifested mental state (Shai and Fonagy, 2014). It is suggested that such interactions would indeed lead the infant to feel safe, to enjoy him or herself, and be available to learning. Therefore, we recommend that book sharing programs also highlight the importance of the relational aspects unfolding in this intimate dyadic interaction, and specifically, on how to appreciate the infant’s mind as this is expressed through bodily movements and respond to it accordingly.

More broadly, the findings from this study speak to the importance of including PEM in clinical interventions designed to enhance and support the quality of parental care. There is growing evidence showing the effectiveness of parental sensitivity enhancement programs to child development and functioning (Bakermans-Kranenburg et al., 2003; Bick and Dozier, 2013). Nonetheless, there appears to be merit in also including an embodied approach to increase parental sensitive and responsive care. Since the non-verbal communication is so predominant and powerful in the parent-infant relationship, we recommend supporting parents by helping them treat and respond to the infant as a psychological agent on an embodied interactive level. This practice of PEM in the clinical setting thus allows the parent to explore what works for this unique parent–infant dyad, and equally important, what might work even better (Shai and Belsky, 2017). The combination of both approaches – verbal and non-verbal – may result in a more pronounced shifts in the quality of parent-infant interactions.

## Conclusion

The present study focused on the longitudinal impact of parental sensitivity and mentalizing on infant cognitive and language development while using mixed methods, including interviews, observations, questionnaires, and objective assessments. Findings revealed that PEM marginally predicted cognitive development and significantly predicted language development 12 months later, over and above parental EA sensitivity, CIB attunement, parental reflective functioning, maternal depression, maternal ethnicity, education, group, and number of children. These findings need to be handled with caution given the high-risk nature of the sample and its small size. Nonetheless, our findings indicate that there parental embodied mentalizing may be important in the establishment of epistemic trust and hold significant value in exposing and discovering the conceptualization and measurement of parental mentalizing beyond the linguistic, declarative domain to include non-verbal, body-based aspects of this parental capacity.

## Data availability statement

The data analyzed in this study is subject to the following licenses/restrictions: Confidentiality of the participants being a clinical sample. Requests to access these datasets should be directed to PF, p.fonagy@ucl.ac.uk.

## Ethics statement

The studies involving human participants were reviewed and approved by the National Health Service REC (Reference: 05/Q0511/47). Written informed consent to participate in this study was provided by the participants’ legal guardian/next of kin.

## Author contributions

DS: conceptualization, methodology, formal analysis, and writing – original draft. AB: conceptualization, formal analysis, and writing – original draft. RS and TN: conceptualization and writing – review and editing. MS and TB: data curation and writing – review and editing. PF: conceptualization, data curation writing – review and editing, and funding acquisition. All authors contributed to the article and approved the submitted version.
